# Decrease in Aflatoxin M_1_ Concentration in Milk during Cholesterol Removal by Application of β-Cyclodextrin

**DOI:** 10.3390/toxins14060379

**Published:** 2022-05-29

**Authors:** Peter Šimko, Lukáš Kolarič

**Affiliations:** Institute of Food Science and Nutrition, Faculty of Chemical and Food Technology, Slovak University of Technology in Bratislava, Radlinského 9, 812 37 Bratislava, Slovakia; lukas.kolaric@stuba.sk

**Keywords:** aflatoxin M_1_, milk, cholesterol, β-cyclodextrin, food safety, mitigation procedure

## Abstract

Approximately one-third of humankind is chronically exposed to the carcinogenic aflatoxin M_1_ contained in milk. As β-cyclodextrin is frequently used in the food industry, its effect on aflatoxin M_1_ concentration was investigated during cholesterol removal from milk due to the similarity among the physicochemical properties of aflatoxin M_1_ and cholesterol. Moreover, the elimination of cholesterol using β-cyclodextrin has been successfully applied in many studies without any substantial effect on the quality of the treated milk. Therefore, milk samples were spiked with aflatoxin M_1_ within the range from 0.20 to 2.00 µg/kg, and cholesterol removal was carried out by 2.0% (*w/w*) β-cyclodextrin addition, as this concentration is enough for the sufficient removal of cholesterol. It was found that the mean cholesterol concentration decreased by 92.3%, while the aflatoxin M_1_ concentration decreased to 0.53 ± 0.04 µg/kg, i.e., by 39.1% after treatment (n = 2). This mitigation procedure itself is easy and inexpensive and thus is fully applicable with a high potential for complete decontamination of aflatoxin M_1_ milk. This method will therefore considerably improve the food safety issues associated with aflatoxin M_1_ presence in milk and dairy products.

## 1. Introduction

Mycotoxins are secondary metabolites produced naturally by molds, and they frequently contaminate food and feed. It is expected that more than 25% of the world’s agricultural production is contaminated with mycotoxins above the EU and Codex Alimentarius limits [1]. One of the most dangerous mycotoxins is aflatoxin B_1_ (AFB_1_), which is produced by the action of *Aspergillus flavus* and *Aspergillus parasiticus* during the production, harvest, storage, and food processing, and it is considered by the US Food and Drug Administration (FDA) an unavoidable contaminant of foods with various serious adverse health effects in humans, such as acute illness and death, liver cancer, nutritional interference [2], and immunologic suppression [3]. After contaminated food/feed is consumed, AFB_1_ is metabolized to aflatoxin M_1_ (AFM_1_) in the liver and subsequently excreted into the milk of lactating humans/animals [4]. In vivo genotoxic tests in *Drosophila melanogaster* revealed that AFM_1_ is three times less dangerous than AFB_1_ in its ability to damage DNA, but its genotoxic effect is compatible with AFB_1_ [5]. Due to these adverse effects, some countries have limited the maximum acceptable limits for AFM_1_ in milk; for example, the FDA sets a limit of 0.5 µg/kg [6] in the USA, while the limit in the EU is 0.05 µg/kg for adults’ food and 0.025 µg/kg for infants’ foods [7].

Milk is highly nutritious and contains many macro- and micronutrients that are essential for the growth and maintenance of human health, especially infants, children, and older adults [8]. According to the Food and Agricultural Organization report, the mean milk consumption per capita in the world is calculated at approximately 100 kg/year; however, it is variable from country to country [9]. Dairy is expected to be the fastest growing livestock sector in the next decade, with global milk production projected to increase by 22%. Increased dairy production will be driven by expanding yields due to the optimization of milk production systems, improved animal health, better genetics and improved feeding efficiencies, and expansions in the inventory of milking animals. The increase in production will be largely supported by the consumer demand for fresh dairy products, particularly in Asian countries. India and Pakistan are expected to account for more than 30% of global milk production by 2030 [10]. 

AFM_1_ is a frequent task of scientific activity, as it is the subject of many published articles. For example, 779 records in the Web of Science Core Collection and 883 records in Scopus databases can be found of research conducted in the last 10 years on findings, risk assessment, and mitigation strategy of AFM_1_ presence in milk and dairy products [11,12,13,14]. To illustrate, some findings of AFM_1_ in milk around the world are briefly summarized in Table 1. According to a worldwide systematic review and meta-analysis [12], the average AFM_1_ concentration in raw and pasteurized milk was 0.057 µg/kg and 0.085 µg/kg, respectively, while the lowest and highest concentrations of AFM_1_ in pasteurized milk were in the goat and cow, respectively. According to Roila et al. [13], the study on the occurrence of AFM_1_ in milk in Italy over the years 2014–2020 revealed that the mean concentration of AFM1 in cow’s milk ranged from 0.009 to 0.015 µg/kg and in ewe’s milk from 0.009 to 0.013 µg/kg. 

It can be seen that, in addition to what is detected various types of milk, the results of the ratios of the number of samples to the number of positive samples, as well as the AFM_1_ concentration range, vary among the countries where there are reported.

In line with the finding of AFM_1_ in food products, great effort has been devoted to the procedures to eliminate AFM_1_ from food products. Considering that AFM_1_ contamination of foods is a great threat to human health and national/international food trade, many studies have been carried out to find efficient detoxification methods. Until now, physical approach (e.g., thermal decomposition, cold plasma, and pulsed light), chemical procedures (e.g., acid/alkali treatment, ozonation, and ammoniation) and biological degradations (e.g., enzymatic degradation and biotransformation) are the three most important detoxification strategies [26]. However, not all AFs can be truly eliminated, nor are all decontamination procedures efficient enough. In addition, some of them are not even applicable in a matrix due to the content of highly reactive and labile compounds. In such cases, the decontamination effect is strongly diminished by the formation of serious nutritional, organoleptic, and technological defects that considerably limit the acceptability of the treated foods on the food market [27,28]. 

β-cyclodextrin (β-CD) is a torus-shaped oligosaccharide made up of α-(1,4) linked seven glucose units, obtained from starch degradation by the enzyme cyclodextrin glucosyltransferase. The β-CD ring is a conical cylinder of an amphiphilic nature, with a hydrophilic outer part (formed by the hydroxyl groups) and a predominantly lipophilic cavity. Both inorganic and organic salts and neutral (nonpolar) molecules can form complexes with β-CD so-called ‘inclusion complexes’. Today, β-CD is frequently used in the food industry for various purposes, e.g., the stabilization of labile compounds, the controlled release of volatile compounds, the elimination of undesirable tastes and odors, dietary fiber food enrichment, and finally, the elimination of cholesterol (CHO) from milk during the production of low cholesterol dairy products. It is notable that the elimination of CHO from milk by β-CD is a ‘soft’ procedure, which means that it does not significantly affect the organoleptic profile of the treated milk [29]. With original organoleptic profiles, these products are therefore highly valued alternatives for consumers, as long-lasting over-limited intake of CHO leads to the development of cardiovascular diseases (CVD), while the consumption of low-cholesterol foods can decrease the total intake, resulting in a lowered incidence of CVD, which brings considerable health benefits to consumers [30]. 

Since the physicochemical properties of AFM_1_ and CHO are similar, the same physicochemical interactions with β-CD can be expected. The most important characteristics that are essential for the formation of an inclusion complex are the size, charge, and polarity of the guest molecule, the effect of the reaction medium, and the temperature. As AFM_1_ and CHO are neutral molecules with a similar molecular weight (328.27 g/mol and 386.7 g/mol, respectively), which are both freely soluble in chloroform and methanol, the formation of inclusion complex AFM_1_-β-CD can therefore be envisaged [31,32]. Hence, this study aimed to investigate the possibility of the elimination of AFM_1_ from milk during CHO removal by application of β-CD.

## 2. Results and Discussion

**Figure 1:**
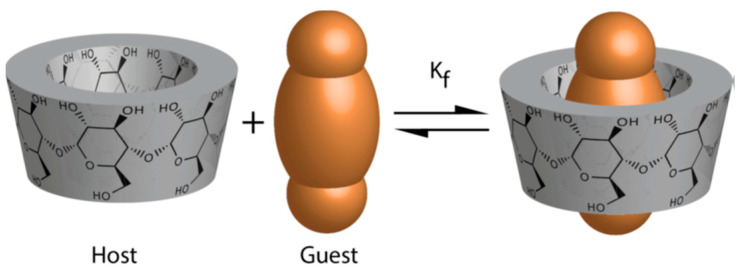
Schematic illustration of the association of the free β-CD (‘host’) and the substrate (‘guest’) to form a substrate—β-CD inclusion complex. Reprinted with permission from Crini, G. A history of cyclodextrins. *Chem. Rev.* **2014**, *114*, 10940–10975. Copyright © (2014) American Chemical Society.

β-CD has special properties, which results in the formation of the so-called inclusion complex, especially with nonpolar compounds. This means that the core of its blunted cone structure forms a dimensionally stable hydrophobic cavity that can trap or encapsulate predominantly nonpolar molecules, including CHO and AFM_1_. The structure of the inclusion complex formation is shown in Figure 1.

In general, the formation of inclusion complexes includes five elementary steps [33]: (I) the substrate approaches the β-CD molecule; (II) the guest molecule becomes released from the layer of water; (III) the guest molecule enters the cavity, and the complex formed is stabilized by van der Waals forces and/or hydrogen bonds; (IV) the expelled water molecules are rearranged and form hydrogen bonds between each other; and (V) the structure of the water is restored around the part of the substrate that remains in contact with the solvent and is integrated into the hydration shell around the β-CD. Finally, the intermolecular and intramolecular hydrogen bonds cause conformational changes that lead to a general thermodynamic stabilization of the inclusion complexes. This description is applicable only for inclusion complexes that are formed in solution [34]. One of the most frequent practical applications of the formation of inclusion complexes is the removal of CHO from milk when the formation of the CHO-β-CD inclusion complex provides a fundamental basis for the production of functional low-cholesterol food products to protect consumers’ health against long-lasting high daily intake of CHO from milk and dairy products [35]. The same situation was also observed in these experiments, as shown in columns A, B, and C of Table 2, when CHO concentrations effectively decreased in all treated samples and the average decrease in CHO concentration was 92.3%. 

However, the data related to the removal of AFM_1_ are much more interesting, i.e., the data obtained when the concentrations of AFM_1_ also decreased during the experiments in all samples and the average concentration decrease in AFM_1_ was 39.1%, as follows from the columns A*, B*, and C* of Table 3. In general, the measure of removal of a contaminant can be expressed by the distribution coefficient *δ* given by the ratio expressed in Equation (1) [36]. In this case, the higher the *δ* value, the more AFM_1_ was removed from milk by the formation of the inclusion complex AFM_1_-β-CD. The values of *δ*_AFM1_ are listed in column D* of Table 3. When comparing the average value of *δ*_AFM1_ with the average value of *δ*_CHO_ (column D of Table 2), it can be seen that CHO was removed more than 18 times more efficiently than AFM_1_. This could be due to the fact that experimental conditions (amount of β-CD addition, time and speed of mixing, temperature, settling time, and speed of centrifugation) were never optimized for the removal of AFM_1_. The values of the distribution coefficients (D column of Table 2 and D* column of Table 3) were tested using the Kolmogorov–Smirnov test to find the measure of the distribution of the calculated data.

Although the test confirmed the normal distribution of the values in the case of δ_CHO,_ in the case of δ_AFM1_, the normal distribution of the values was not confirmed. This finding can be associated with the fact that AFM_1_ concentration is, in general, five orders lower than the CHO concentration, and at this AFM_1_ concentration level, optimal conditions of AFM_1_-β-CD inclusion complex formation were not adjusted. In addition, the results of the Kolmogorov–Smirnov test also suggest that the formation of the AFM_1_-β-CD inclusion complex may be affected by the presence of other (unknown) compounds that appeared accidentally in individual milk samples and were able to compete for β-CD molecules at the µg/kg concentration level. In addition, the correlation analysis between distribution coefficients (values in D and D* columns) confirmed a positive correlation with *r* = 0.83. To find statistically significant differences in the changes in CHO concentration, one-way analysis of variance (ANOVA) and the Tukey comparison test were used, while in the case of AFM_1_ concentration, the nonparametric Wilcoxon test was applied. The tests confirmed statistically significant differences between the initial and the final concentrations of both CHO and AFM_1_ compounds. Therefore, the obtained results are a promising basis for further adjustment and optimization of the AFM_1_ removal procedure parameters to achieve complete AFM_1_ removal from the milk matrix. The application of the procedure itself is easy, safe, effective, and low in cost and labor, with no substantial negative effects on nutritional, organoleptic, or technological parameters of milk or dairy products, which also confirms its current usage in the dairy industry, although for different purposes, that is, for the removal of CHO [35,36]. In addition, the application of the procedure can effectively prevent economic losses associated with frequent findings of overly limited AFM_1_ concentrations in traded dairy products [37]. 

Compared to other approaches to AFM_1_ mitigation in milk, some studies have shown the importance of the prevention of crop contamination in pre-harvest and post-harvest stages, and other studies proposed direct methods to reduce AFM_1_ in milk [38]. For example, Hassanpour et al. [39] described a procedure for the reduction of AFM_1_ concentration in pasteurized milk using low-dose gamma irradiation. The average reduction rate of AFM_1_ was 55.1 and 99% after 4 and 8 days, respectively. Recently, Chaudhary and Patel [40] presented an interesting approach to remove AFM_1_ from milk by isolated lactic acid bacteria. In addition, Kuhari et al. [41] noticed that the efficiency of AFM_1_ decontamination by lactic acid bacteria ranged from 21 to 95% and stated that this procedure should not influence the final organoleptic properties of dairy products. However, the use of microbial decontamination has also some limitations, e.g., the addition of microbial agents to milk is acceptable only to a certain limit, so other additional treatment for their removal/devitalization could be required. Moreover, microorganisms release enzymes, the activity of which can negatively affect fat and protein quality or lactose content. Finally, the binding of AFM_1_ to microbial adsorbents is partially reversible [42]. Some other absorbents were also tested to mitigate AFM_1_ concentration in milk, e.g., sodium bentonite (64.5% effectivity in reduction), calcium bentonite (31.4% effectivity in reduction), mycosorb (58.5% effectivity in reduction), and activated charcoal (5.4% effectivity in reduction) [38]. However, the use of such compounds in food technology is questionable and can alter the organoleptic and nutritional value of the final products. Therefore, the removal of AFM_1_ in the dairy industry remains open due to a lack of a ‘fine and friendly’ procedure that does not affect the nutritional, organoleptic, and technological parameters of dairy products [43]. 

## 3. Conclusions

Due to ingestion of AFB_1_-contaminated feed, lactating animals secrete its AFM_1_ metabolite into milk. Therefore, dairy products, such as milk, cheese, and yogurts, are frequently contaminated with this toxin, presenting serious health implications for consumers and economic losses in trading due to over-limited AFM_1_ concentrations. Therefore, the effective removal of AFM_1_ from milk is a crucial factor that can positively affect food safety issues and problems associated with the handling of contaminated products. In this article, a method for the removal of AFM_1_ from milk has been developed based on physicochemical interaction in which β-CD forms inclusion complex AFM_1_-β-CD, which is then easily separated from milk by centrifugation. As shown in the experiments, the average removal efficiency of AFM_1_ was observed at a level of 39.1%. The procedure of removal of AFM_1_ with β-CD was not studied before, so these first findings show a great possibility of a novel decontamination step. The procedure itself is instantly applicable, since β-CD is currently used in the dairy industry for the removal of CHO. Therefore, this method could help resolve the health problems associated with the chronic presence of AFM_1_ in milk and dairy products around the world and the tasks associated with handling over-limited concentrations of AFM_1_ in traded dairy products. Future work should focus on optimizing the steps of the processing conditions for AFM_1_, as these were not evaluated. 

## 4. Materials and Methods

### 4.1. Samples

Seven brands of commercial pasteurized cow’s milk (3.5% declared fat content in the samples 1; 2; 4; 5; 6; and 7, sample no. 3—4.0% declared fat content) were bought in a local market in Bratislava, Slovak Republic. 

### 4.2. Chemicals

Beta-cyclodextrin was purchased from Wacker Chemie AG (Burghausen, Germany, ≥95.0%), and cholesterol of analytical standard grade and aflatoxin M_1_ (analytical standard 0.5 μg/mL in acetonitrile) of analytical standard grade were purchased from Merck, KGaA (Darmstadt, Germany). Chloroform, n-hexane, ethanol 96%, and anhydrous Na_2_SO_4_ p.a. grade were bought at Centralchem Ltd., (Bratislava, Slovak Republic). KOH p.a. grade was supplied by Mikrochem Ltd., Pezinok, Slovak Republic), and both methanol and acetonitrile of HPLC grade were provided by Fisher Chemical Ltd., (Loughborough, UK). 

### 4.3. Instruments

HPLC system 1260 Infinity (Agilent Technologies, USA) was composed of a vacuum degasser, quarterly pump, autosampler, UV-DAD detector, FD detector, analytical column Zorbax Eclipse Plus C_18_ (2.1 × 50 mm, 5 µm particle size), and guard column Zorbax SB-C_18_ (4.6 × 12.5 mm, 5 µm particle size). PTFE filters with 0.2 μm membrane (Agilent, Santa Clara, CA, USA) were used. For the purposes of sample preparation, a rotary vacuum evaporator (Heidolph, Germany), centrifuge (Hettich Zentrifugen, Germany), magnetic stirrer (Arex-6 Connect Pro, Velp Scientifica, Italy), and an analytical balance (Sartorius, Goettingen, Germany) were applied.

### 4.4. Experiments

The samples were divided into two groups. The first one was analyzed for CHO and AFM_1_ concentrations. These analyses confirmed native AFM_1_ concentrations under LOQ equal to 0.013 µg/kg. The second group was spiked with AFM_1_ to obtain initial concentrations in milk of 0.20, 0.40, 0.60, 0.80, 1.00, 1.20, and 2.00 µg/kg. Then, the samples were treated with β-CD to remove CHO and AFM_1_. After the treatment procedure, the samples were analyzed for CHO or AFM_1_ concentration.

### 4.5. Treatment of Milk for Removal of CHO and AFM_1_

The samples were treated with optimized conditions as described previously [30]: 250 g of milk was placed in a beaker, and 2.0% of β-CD was added. The mixture was stirred at 840 rpm using a magnetic stirrer for 10 min at 25 °C, and then the treated milk was left static for 120 min at 4 °C and centrifuged at 130× *g* for 20 min. After centrifugation, the milk supernatant was analyzed for CHO and AFM1 concentration. The number of samples for both groups was 7.

The measure of removal was calculated and expressed as distribution coefficients δ according to the Equation (1)

(1) $$\delta =\frac{c_{0}\;-\;c_{\infty }}{c_{\begin{array}{c} \infty \end{array}}}$$

where *c*_0_ is the initial concentration and *c*_∞_ is the equilibrium (final) concentration of the contaminant [44].

### 4.6. Preparation of Milk for CHO Analysis

The samples (n = 7) were prepared [45,46] as follows. First, 5.0 g of milk was refluxed with 15 mL of 1 mol/L methanolic solution of KOH for 15 min. Then, the cooled matter was extracted twice with a mixture of n-hexane and chloroform (1:1, *v/v*) to obtain 15 mL of total extract. To increase the polarity of the saponifiable residue, 10 mL of deionized water was added. To avoid the formation of emulsion during extraction, 1 mL of ethanol (96%) was added to the saponified matter. Then, the extract was filtrated through anhydrous Na_2_SO_4_ and evaporated using a rotary vacuum evaporator until it was dry; the residue was dissolved in 3 mL of methanol, filtered using syringe PTFE filter with 0.2 μm membrane, and analyzed by HPLC. 

### 4.7. HPLC Determination of CHO Concentration

HPLC was performed according to [45,46], using an isocratic elution at a flow rate of 0.5 mL/min with a mobile phase composed of acetonitrile/methanol 60:40 (*v/v*). The injection volume was 10µL, and the temperature was set at 30 °C. Zorbax Eclipse Plus C18 column (2.1 × 50 mm, 5 µm particle size, Agilent) was used as a stationary phase with the guard column Zorbax SB-C18 (2.1 × 12.5 mm, 5 µm particle size, Agilent). At these conditions, CHO was eluted within 2.2 min of the analysis, and the detector was operated at 205 nm. Data were recorded and treated using the OpenLab CDS software, ChemStation Edition for LC, and LC/MS systems (product version A.01.08.108). All determinations were carried out in duplicate. 

### 4.8. Preparation of Milk for AFM_1_ Analysis

A sample treatment (n = 7), based on the AOAC method [47] and modified by [18], was carried out as follows: 50 g of milk, previously skimmed by centrifugation at 3700× *g* for 15 min, was loaded on immuno-affinity chromatography column (R-Biopharm AG, Darmstadt, Germany)) and washed with 50 mL water. Then, the analyte was eluted with 2 mL acetonitrile–methanol mixture (60:40 *v/v*), eluent evaporated to near dryness, residue dissolved with 200 µL acetonitrile–methanol mixture (60:40, *v/v*) plus 200 µL water, and finally, filtered on a 0.2 µm membrane filter. 

### 4.9. HPLC Determination of AFM_1_ Concentration

HPLC was performed according to [18] using an isocratic elution at a flow rate of 1 mL/min mobile phase composed of a water–acetonitrile–methanol mixture (65:15:20, *v/v*/v). The injection volume was 10µL, and the temperature was set at 30 °C. In these conditions, AFM1 was eluted in 4.5 min of analysis, and the fluorescence detector operated at an excitation wavelength of 360 nm and an emission wavelength of 430 nm. Data were recorded and treated using the OpenLab CDS software, ChemStation Edition for LC, and LC/MS systems (product version A.01.08.108). All determinations were carried out in duplicate.

### 4.10. Validation of Analytical Procedures

The method for the determination of the CHO concentration in milk and other dairy products was validated using an *in-house* regime [45,46]. The method for the determination of the AFM_1_ concentration met the validation criteria (LOD, LOQ, recovery, ruggedness, repeatability, and linearity) set by European Commission [48].

### 4.11. Statistical Analysis

Results are expressed as mean ± standard deviation, n = 2. Statistical analysis was performed using the XLSTAT tool of Microsoft Excel 365 (version 2012, Microsoft, Redmond, Washington, USA). The obtained data were subjected to one-way analysis of variance (ANOVA), and Tukey’s comparison test, and the values were considered significantly different when *p* < 0.05. Additionally, Kolmogorov–Smirnov and Wilcoxon nonparametric tests for data treatment were used.

## Figures and Tables

**Table 1:** AFM_1_ findings in milk in some countries around the world.

Sample	No. of Samples/No. of Positive Samples	Concentration Range of AFM_1_ (µg/kg)	Country	Source
Raw milk Pasteurized milk UHT milk	105/75 15/15 15/15	0.005–0.198 0.017–0.187 0.012–0.146	Bangladesh	Sumon et al. [15]
Fresh milk	52/21	0.01–3.385	Brazil	Goncalves et al. [16]
Pasteurized and UHT milk	242/178	0.001–0.352	China	Xiong et al. [17]
Raw milk	1668/36	0.01–0.208	Italy	Bellio et al. [18]
Bovine milk Buffalo milk	375/154 170/70	0.01–9.18 0.01–6.41	India	Pandey et al. [19]
Raw milk	290/145	Nd *–8.35	Mexico	Carvajal et al. [20]
Bovine milk Goat milk	29/29 87/41	up to 0.081 up to 3.108	Nigeria	Akinyemi et al. [21]
Fresh milk	107/76	0.004–0.845	Pakistan	Iqbal et al. [22]
Raw milk	150/150	0.01–1.2	Serbia	Kos et al. [23]
Raw milk	100/45	0.02–0.08	South Korea	Lee et al. [24]
Fresh milk	44/42	0.22–6.90	Sudan	Elzupir et al. [25]

Nd *—not detected.

**Table 2:** Effect of β-CD treatment on the concentration of CHO in milk.

	A	B	C	D
Sample No.	Initial Concentration of Cholesterol (mg/kg) ^a^	Concentration of Cholesterol after Removal (mg/kg) ^a^	Measure of Cholesterol Removal (%)	Distribution Coefficient δ_CHO_
1	129.04 ± 2.13	10.36 ± 2.11 ^+^	92.0	11.46
2	135.78 ± 6.01	6.47 ± 1.59 ^+^	95.2	19.99
3	150.39 ± 0.64	5.25 ± 0.03 ^+^	96.5	27.65
4	113.32 ± 6.30	8.92 ± 0.02 ^+^	92.1	11.70
5	123.01 ± 2.21	1.43 ± 0.63 ^+^	98.8	85.02
6	103.92 ± 0.43	9.47 ± 0.21 ^+^	90.9	9.97
7	122.33 ± 1.45	23.49 ± 1.50 ^+^	80.8	4.21
Average	125.40 ± 2.74	9.34 ± 0.87 ^+^	92.3	24.28

^a^ The results are expressed as mean ± standard deviation (n = 2). ^+^ Statistically significant difference at *p* < 0.05.

**Table 3:** Effect of β-CD treatment on the concentration of AFM_1_ in milk.

	A*	B*	C*	D*
Sample No.	Concentration of AFM_1_ after Spiking (µg/kg)	Concentration of AFM_1_ after Removal (µg/kg) ^a^	Measure of AFM_1_ Removal (%)	Distribution Coefficient δ_AFM1_
1	0.20	0.13 ± 0.06 ^+^	35	0.54
2	0.40	0.25 ± 0.02 ^+^	38	0.60
3	0.60	0.36 ± 0.02 ^+^	40	0.67
4	0.80	0.47 ± 0.03 ^+^	41	0.70
5	1.00	0.55 ± 0.04 ^+^	45	0.82
6	1.20	0.80 ± 0.04 ^+^	33	0.50
7	2.00	1.16 ± 0.06 ^+^	42	0.72
Average	0.89	0.53 ± 0.04 ^+^	39.1	0.68

^a^ The results are expressed as mean ± standard deviation (n = 2). ^+^ Statistically significant difference at *p* < 0.05.
